# From the sticky floor to the glass ceiling and everything in between: protocol for a systematic review of barriers and facilitators to clinical academic careers and interventions to address these, with a focus on gender inequality

**DOI:** 10.1186/s13643-020-1286-z

**Published:** 2020-02-10

**Authors:** Jennifer V. E. Brown, Paul E. S. Crampton, Gabrielle M. Finn, Jessica E. Morgan, on behalf of the project team

**Affiliations:** 1 Centre for Reviews and Dissemination, University of York, York, UK; 2 Health Professions Education Unit, Hull York Medical School, University of York, York, UK; 3 Hull York Medical School, University of York, York, UK

**Keywords:** Doctor, Dentist, Clinical academic, Careers, Integrated academic training, Gender inequality, Equity, Diversity and inclusion, Culture change, Systematic

## Abstract

**Background:**

Gender inequality within academic medicine and dentistry is a well-recognised issue, but one which is not completely understood in terms of its causes, or interventions to facilitate equality. This systematic review aims to identify, critically appraise, and synthesise the literature on facilitators and barriers to progression through a clinical academic career across medicine and dentistry. It will also explore interventions developed to increase recruitment and retention to clinical academic careers, with a particular focus on gender inequality.

**Methods:**

The search will cover five databases (MEDLINE (including MEDLINE Epub Ahead of Print, MEDLINE In-Process & Other Non-Indexed Citations, and MEDLINE Daily), Cochrane Controlled Register of Trials (CENTRAL), PsycINFO, and Education Resource Information Center (ERIC)), reference lists, and forward citation searching. We will include studies of doctors, dentists, and/or those with a supervisory role over their careers, with or without an academic career. Outcomes will be study defined, but relate to success rates of joining or continuing within a clinical academic career, including but not limited to success in gaining funding support, proportion of time spent in academic work, and numbers of awards/higher education qualifications, as well as experiences of professionals within the clinical academic pathway. Study quality will be assessed using the Cochrane risk of bias tool for randomised controlled trials, the Newcastle-Ottawa tool for non-randomised studies, and the QARI tool for qualitative studies. Detailed plans for screening, data extraction, and analysis are provided within this protocol.

**Discussion:**

This systematic review is situated within a larger project evaluating gender inequalities in clinical academic careers. This review will identify and synthetize barriers, facilitators, and interventions addressing gender inequalities in clinical academia. Our findings will increase awareness of inequalities in clinical academic careers through informing clinical academics, regulators and funders of the issues involved, and potential interventions to counteract these. Results will be published in a peer-reviewed journal.

**Systematic review registration:**

Open Science Framework: https://osf.io/mfy7a

## Background

In the UK, HEE’s Integrated Academic Training (IAT) programme provides a strategic framework for the development of clinical academics: talented, research-focussed, and expert doctors and dentists who will bring additional skills into the NHS for the benefit of patients. The reasons why some doctors and dentists decide to pursue a clinical academic career and others do not are seldom researched and, consequently, poorly understood. In recent years, there have been concerns that the clinical academic career pathway fosters inequality by being designed in a way that supports certain groups of doctors/dentists but not others. Research to inform future strategies to ensure that the programme is fair for all begins with an understanding of the current situation.

Due to the relatively small numbers of clinical academics, an understanding of academic medicine and dentistry needs to be informed by data from the medical and dental professions as well as academia itself. While there is still a gender imbalance in favour of men in the number of registered doctors, NHS Workforce Statistics data show a trend that this difference has reduced slowly over the past decade. In March 2019, 45% of all doctors registered in England were women [1]. The gender imbalance in academic medicine, however, is still rather more pronounced and particularly prominent at senior levels [2, 3]. Data from the Medical Schools Council show that only 26.3% of clinical academics are female [4]. The imbalance increases substantially through the ranks with female lecturers accounting for 42.3%, dropping to 15.6% of Professors. The same report found that there are gender differences in age and clinical academic grade, which imply that men are more likely to achieve senior levels at a younger age. Evidence shows women in academia are awarded less grant funding and have fewer high-impact publications, which are key factors in progressing their academic careers [5–8]. An NIHR-funded project published in 2019 illustrated that men and women report different experiences of research culture. Across a range of dimensions, men appeared to have a more positive view than women [9].

The ‘glass ceiling’ is well documented in academia [10–12] and is described as an invisible barrier to advancement in a profession beyond a certain level in a hierarchy, especially affecting women and members of minorities. Although the ‘glass ceiling’ is a metaphor describing an inequitable architectural feature of career paths, its potential impact on individuals is profound. Despite increased awareness across academia, medicine, as well as industry and society more widely, many women continue to struggle to reach career and salary positions equal to their male colleagues, even when they have comparable skills and experience. The persistent barriers that contribute to the glass-ceiling effect within academic medicine include traditional gender roles, preferential treatment of male candidates, structures that are unsupportive of family-related career breaks, and a lack of effective mentors who would be ideally placed to champion female academics’ ambitions [13].

More recently, the medical literature has been using the term ‘sticky floor’ which describes the position of women in academic medicine and dentistry where fewer are promoted and fewer are given any institutional resource at the start of their careers to set them on their way, when compared with their male colleagues [10, 13]. The metaphor gained traction following research that surveyed male and female medics who began their roles at the same time. Women were not only faced by the ‘glass ceiling’ in terms of promotion, but were ‘stuck to the floor’ by the lack of investment [14].

Interventions to address gender inequality in academic medicine and dentistry have generally been poorly described. In academia itself, the Athena SWAN (Scientific Women’s Academic Network) charter, established in 2005, requires institutions to demonstrate their performance in a number of predefined areas, focusing on the advancement of women’s careers. The need for universities and departments to achieve higher levels of Athena SWAN award has been cemented by the fact that funding bodies such as the NIHR will only award certain grants to institutions who have achieved a silver award. In 2016, AdvanceHE undertook research funded by Wellcome to showcase best practice initiatives to tackle gender inequalities across the UK [15].

Meanwhile, in 2018, a team based in Australia concluded that targeted interventions can be effective in supporting women’s careers in academic medicine and dentistry, among other disciplines. However, their systematic review also revealed that it is important how these interventions are delivered. Bottom-up approaches, which place the onus largely on the individual wishing to progress their career, were less successful [16]. Our review aims to expand on these findings by using a more comprehensive search strategy and taking into account discrimination and biases based on characteristics other than gender, as well.

This systematic review is situated within a larger project that has a view to identifying possible mechanisms that perpetuate the current imbalanced gender profile in academic medicine and dentistry and also aims to identify and appraise existing interventions that address these issues. Specifically, the systematic review will evaluate the existing evidence on factors that promote or hinder progression in academic medicine and dentistry, interventions, and attrition in clinical academic careers. It will be a mixed-methods review focused on medical and dental professions and will evaluate and summarise a wide breadth of literature within the area. There are scoping elements to the review, followed by deeper evaluation and synthesis. While acknowledging that inequality is not exclusively about gender, the focus will be on gender differences with the aim of identifying strategies to increase the proportion of female clinical academics across all levels/grades and to support the progression of female candidates beyond post-graduate level. The review will provide the foundation for our linked primary qualitative research study (in-depth interviews and audio-diaries).

## Objectives

This systematic review aims to:

(1) Identify, critically appraise, and synthesise the literature on facilitators and barriers to progression through a clinical academic career across medicine and dentistry

(2) Identify, critically appraise, and synthesise research on existing interventions developed to increase recruitment, retention, and progression to clinical academic careers.

We intend to shed light on barriers and facilitators to clinical academic careers for both men and women. Once we have a clearer understanding of the overall picture, we will shift our focus onto any potential gender differences in the identified supportive or hindering factors, in line with the overarching project.

## Methods

This systematic review is situated within a larger project that aims to identify possible mechanisms that perpetuate the current imbalanced gender profile in academic medicine and dentistry and also aims to identify and appraise existing interventions that address these issues, as outlined within the introduction to this review.

The present review protocol has been registered within the Open Science Framework (registration number: osf.io/mfy7a) and is being reported in accordance with the reporting guidance provided in the Preferred Reporting Items for Systematic Reviews and Meta-Analyses Protocols (PRISMA-P) statement [17] (Additional file 1).

### Search and information sources

The following relevant database will be searched for studies: MEDLINE (including MEDLINE Epub Ahead of Print, MEDLINE In-Process & Other Non-Indexed Citations, and MEDLINE Daily), Cochrane Controlled Register of Trials (CENTRAL), PsycINFO, and Education Resource Information Center (ERIC) database. The search strategy will include subject headings and free-text terms for clinical academics. A date limit will be applied to the strategy to restrict retrieval to studies published from 2004 onwards, reflecting the era of the Athena SWAN initiative. The full search strategy for Ovid MEDLINE can be found in Additional file 2. This strategy will be translated to run appropriately on the other databases.

As preliminary searches using the strategy in Additional file 2 retrieved a very high number of potentially eligible records, we developed a narrower search strategy on the same databases in an attempt to limit the number of hits. This strategy is included in Additional file 3. The results from this narrower search (which our pilot work found contains a higher proportion of potentially eligible records) as well as 1000 random records from the main search will be used to ‘train’ the text mining algorithm in the reference management software, Rayyan (further details provided in the ‘Data management and selection process’ section).

Reference lists of relevant systematic reviews and included articles will be reviewed, and forward citation searches of key papers will be undertaken. Authors of relevant studies may be contacted as time allows to seek further studies. We will contact the project funders to request any relevant reports or other work within their portfolio. Published and unpublished studies will be sought and no study design restrictions applied. A time limit for eliciting further studies of 3 months will be applied to ensure that the results of the review are available to inform further aspects of the overarching multi-methods research.

### Inclusion and exclusion criteria

Studies will be included in the review if they meet the following criteria:

#### Population

The study population will include doctors, dentists, and/or those with a supervisory role over their careers (e.g. programme directors, deans). Studies which include mixed groups of professionals will only be included if the doctor/dentist group is reported separately, or if they comprise more than 50% of the participants. Studies of qualified doctors and dentists of all specialties and at all levels of career are eligible for inclusion. Those with academic careers can be at any level from pre-doctoral to professor. The review expressly does not include medical and dental students, though future work may wish to explore the various influences on those at such an early career phase. Studies which explore why doctors and dentists have chosen not to undertake a clinical academic career or why they no longer have a clinical academic career (when they previously were following one) will be eligible for inclusion. For the purpose of this review, an academic career refers to those engaged in research, not purely teaching or educational roles.

While there are pathways that offer a clinical academic career to nurses, midwives, and other allied health professionals, this review will focus exclusively on doctors and dentists, consistent with the needs of the funders of the research. This also reflects that the main pathways for clinical academic careers in the UK (funded by the NIHR) separate doctors and dentists from other healthcare professionals.

Given that the funders of the review and the main dissemination targets for the findings are based within the UK, we have mainly searched for the British terms for clinical and academic career pathways. We have not expressly searched for American terminology, or those from other countries; however, if identified by the search, these studies would be eligible for inclusion.

#### Topics of interest

- Factors influencing recruitment and retention to clinical academic careers, including barriers and facilitators. This may include but is not limited to funding, training opportunities, cultural aspects, barriers experienced by underrepresented minorities, issues related to academics with young families, and experiences surrounding roles models.
- Interventions to increase recruitment to clinical academic careers and to improve retention in clinical academic careers. These may include, but are not limited to, specific funding opportunities, training opportunities, development programmes, mentorship programmes, and strategies which specifically aim to increase academic engagement of specific groups, e.g. family-friendly strategies aiming to increase the involvement of women in clinical academia.
- Where multiple barriers, facilitators, and interventions are described within and across studies, each will be extracted and included for analysis within the review.

#### Outcomes

Outcomes will be study defined, but related to success rates of joining or continuing within a clinical academic career, including but not limited to success in gaining funding support, proportion of time spent in academic work, and numbers of awards/higher education qualifications, as well as experiences of professionals within the clinical academic pathway.

#### Study design

Studies will be included from all forms of quantitative and qualitative research provided they inform the research objectives. This will include but not be limited to:

- Quantitative research: randomised controlled trials (RCTs); including quasi-RCTs and cluster-RCTs; observational cohort studies (prospective and retrospective); and studies reporting survey data will be eligible for inclusion within the review.
- Qualitative research: methodologies including ethnography, phenomenology, and grounded theory. Studies that use qualitative methods but which do not state an explicit methodology are also eligible to be included, provided they present qualitative data. This includes, but is not limited to, studies using focus group discussions, interview studies, and observational studies. Similarly, mixed-methods studies are eligible for inclusion if they provided sufficient data.

Studies will be limited to those written in the English language for two reasons. Firstly, these are most likely to reflect the cultural experiences of the group in which we plan to apply the results, that is clinical academics in the UK. Secondly, the benefit of qualitative research is to allow participants to express their experiences and perceptions, the clarity of which could be lost through translation and thus the results of the synthesis could become inaccurate. Furthermore, studies will be limited to those performed in high-income countries, in recognition of the cultural and organisational setting in which the research findings are to be applied.

Studies will be included where they are available in full-text format. Conference abstracts will not be eligible for inclusion. Editorials, letters, and opinion pieces will not be eligible for inclusion.

### Data management and selection process

References will be managed in EndNote X9 [18] and exported into Rayyan [19] for study selection. Initially, we will import records identified from the narrower secondary search strategy which we expect to contain a higher proportion of potentially eligible records. Following this initial training of the software, we will import 1000 random unique records from the wider search (see Additional file 2) into Rayyan. This batch of records will also be screened in its entirety. These titles and abstracts will be screened independently and in duplicate by a core team of reviewers who will liaise closely to ensure consistency in eligibility decisions. This process will ‘train’ the text mining algorithm within Rayyan to recognise and prioritise the most relevant records.

As a final step, we will import all further unique records from the broader search strategy (see Additional file 2). The text-mining algorithm will then automatically prioritise the most relevant records and bring them to the top of the list. To manage workload, at this point, we will bring in reviewers from the wider project team to support the screening process. Once there is good agreement between all reviewers, records will be single screened. We will keep track of the rate of records marked for inclusion for each set of 1000 search results. At least 25% of identified titles and abstracts in the broader search strategy will be assessed. However, if the rate of screening includes has not fallen dramatically from baseline at this point, then we will continue until there is team agreement that the rate of includes has fallen sufficiently. We will further explore the similarity index in Rayyan to ensure that no relevant titles and abstracts have been missed.

Disagreements regarding which studies to include will be resolved by consensus or, if this proves impossible, by recourse to another team member. A similar approach will be taken to screening full texts.

### Data extraction and study quality

Data will be extracted by one researcher using a standardised data extraction form and will be independently checked by a second researcher. The information to be extracted is given in detail in Additional file 4. Broadly, this will include general information, detailed study information, participant details, and outcomes. Qualitative data from research reports will be coded by one researcher and reviewed by other members of the research group. The quality of studies will be assessed using the Cochrane risk of bias tool for RCTs [20], the Newcastle-Ottawa tool for non-randomised studies [21], and the QARI tool for qualitative studies [22]. The components of this quality assessment will be presented in both narrative and tabular form.

### Analysis

Key study characteristics and outcome data will be summarised in narrative and tabular form. An overview of the literature base, including any significant gaps in the current understanding of the issues, will be provided. In the first instance, we will analyse quantitative evidence, i.e. from RCTs and any non-randomised studies, and qualitative evidence separately.

#### Quantitative analyses

Where appropriate, we will combine quantitative data in meta-analyses but we anticipate that we will not have sufficient data to conduct meaningful statistical analyses. Therefore, we will narratively synthesise quantitative evidence on interventions that address facilitators and barriers to clinical academic careers, following suitable techniques outlined in the CRD guidance [23]. We will synthesise data at individual, departmental, and organisational levels, paying particular attention to gender, ethnicity, clinical specialty, primary vs secondary care setting, and academic field (e.g. laboratory-based research, clinical trials, systematic reviews, other research methodologies). If appropriate, we will address these factors in formal subgroup analyses.

Similarly, sensitivity analyses will be performed where appropriate, including but not limited to, location of study, risk of bias, conference abstract vs full-text articles, and era of publication. Heterogeneity in any quantitative analyses will be explored both narratively and statistically (using *χ*^2^ tests, the *I*^2^, and tau^2^ statistics and by visual inspection of the forest plots). The risk of publication bias will be explored if there are ≥ 5 comparative studies reporting the same outcome using contour-enhanced funnel plots and Harbord and Peters tests [24].

#### Qualitative (narrative) analyses

A framework analysis will be performed which will allow for the integration of findings across the different components of the project, providing triangulation and further understanding of the research project [25]. The qualitative synthesis will be led by one researcher and reviewed with other researchers. Again, analyses will focus on the influence and impact of factors such as gender, ethnicity, clinical specialty, and academic field (e.g. laboratory-based research, clinical trials, systematic reviews, other research methodologies). The conceptual contribution of each study will be explored in relation to the final synthesis. We will also examine the literature base to establish how it is conceptually organised and to investigate whether there is any dominance regarding geography, professional interest, and theoretical standpoints.

#### Combined synthesis

Following individual analyses of quantitative and qualitative evidence, we will draw the two components of the review together to allow comparisons between the different findings and informing further exploration to provide depth to the review. This is a key stage in the overarching project as these findings will inform the primary qualitative research and will be conducted and reviewed in close collaboration of the entire project team. The report will detail the various aspects of the review and the literature development of constructs within this process. The strength of the whole body of evidence will be assessed narratively, taking into account the various aspects of the review, alongside the risk of bias findings.

### Dissemination plan

Any substantial amendments to this protocol will be documented on the Open Science Framework page for this project (https://osf.io/mfy7a).

The results of this systematic review will crucially inform the development of qualitative research as part of the overarching project. As such, findings will be disseminated to qualitative research participants as appropriate to inform their taking part in the interviews and audio-diaries.

Reports will be provided to the funders at the half-way point and upon completion of the work. The final report will also be published. The study team will be working closely with the UK Clinical Academic Training Forum (CATF) and the study funders to ensure that the findings are communicated to those involved in the clinical academic career pathway and its development.

In addition, the systematic review will be submitted for publication in a scientific journal reported according to PRISMA guidelines [26]. We will also submit our findings to relevant conferences for oral or poster presentations (e.g. the Association for the Study of Medical Education (ASME), the Association for Medical Education Europe (AMEE) Annual Meeting). The findings of the systematic review will inform any outputs from the overarching project, including oral presentations, workshops, and seminars.

To increase the accessibility of our work to a wider audience, we will produce blogs/podcasts and maintain an active social media profile ‘Gender Inequalities in Clinical Academic Careers’ (@GenderClinical), sharing findings of the review and their relevance within the dissemination strategy of the overarching project. We will liaise with established initiatives such as Women Speakers in Healthcare (WSH) and the Medical Women’s Federation (MWF), seek collaboration with existing Athena SWAN/Equality & Diversity activity locally, and aim to increase exposure via contribution to high impact (social) media outputs (e.g. Guilty Feminist podcast, The Conversation, BMJ Opinion).

## Discussion

Through the systematic review, the detailed examination and assessment of the existing research base will inform the design, performance, and analysis of the primary qualitative research, providing the team with a deeper understanding of the barriers and facilitators, faced by clinical academics as they progress through their career. Simultaneously, the primary research will feed into the systematic review, providing additional insight and understanding of why some interventions may or may not work to promote recruitment and retention of female clinical academics at key transition points.

Potential limitations of the underlying studies include poorly reported and/or poorly conducted studies, largely from the USA and largely within medicine (rather than dentistry) combined with an anticipated high volume of potentially eligible studies. This will present a challenge in terms of resource and expertise. Our analyses will be carried out with close attention to the quality of the studies involved, so as to determine the impact of lower quality studies on the overarching findings. We will report any influence of poor quality studies and, where this is found, will also report the findings without these studies included. We are an experienced team embedded within a world leading department and at the cutting edge of novel SR methods, such as text mining. Where necessary, we will liaise with the funders and colleagues in the USA to identify comparable training and funding pathways, to facilitate the translation of our findings into the UK context.

We aim to provide practical solutions and considerations to support healthcare and stakeholder organisations, alongside advancing knowledge on the topic through open-access high-impact publications. The work is funded by, and has the support of, a broad range of funders with high levels of influence over clinical academic pathways in the UK and more broadly. Thus, we anticipate significant pathways to impact from the resultant review. Ultimately, this work has the potential to increase awareness of inequalities in clinical academic careers through informing clinical academics, regulators and funders of the issues involved, and potential interventions to counteract these. The work also has the potential to inform policy change, through influencing funders and informing the development of guidelines for Health Education England and others involved in support clinical academic career pathways. We aim to be able to offer advice and support to pilot interventions within health education institutions, with the potential to further develop work streams to implement our research findings, in order to support the aim to increase access to academic medicine and dentistry for under-represented groups. This review is purposefully limited to qualified doctors and dentists; thus, the results will mainly apply to this specific group. However, there are likely to be findings identified, both within the exploration of barriers and facilitators, and within possible interventions, that might also be transferable to the undergraduate population or to nurses, midwives, and other allied health professionals.

## Supplementary information

**Additional file 1.** Preferred Reporting Items for Systematic Reviews and Meta-Analyses Protocols (PRISMA-P) statement.

**Additional file 2.** Full search strategy for Ovid MEDLINE.

**Additional file 3.** Supplementary search strategy for Ovid MEDLINE.

**Additional file 4.** Data extraction form.

## Data Availability

Not applicable (all data referred to in this article will have been obtained through reading original articles or contacting the authors of cited studies).

## Abbreviations

AMEE: Association for Medical Education in Europe
ASME: Association for the Study of Medical Education
CATF: Clinical Academic Training Forum
CRD: Centre for Reviews and Dissemination
HEE: Health Education England
IAT: Integrated Academic Training
MRC: Medical Research Council
NHS: National Health Service
NIHR: National Institute for Health Research
PRISMA: Preferred Reporting Items for Systematic Reviews and Meta-Analyses
RCT: Randomised controlled trial
UK: United Kingdom
WSH: Women Speakers in Healthcare
