# Supplementary material for: From the sticky floor to the glass ceiling and everything in between: protocol for a systematic review of barriers and facilitators to clinical academic careers and interventions to address these, with a focus on gender inequality
Source: Syst Rev. 2020 Feb 10;9:26. doi: 10.1186/s13643-020-1286-z (PMC7011470; doi:10.1186/s13643-020-1286-z)
Supplement: Supplementary file 2 — Additional file 2. Full search strategy for Ovid MEDLINE. [file 13643_2020_1286_MOESM2_ESM.docx]

### Additional file 2: Main search strategy

Database: Ovid MEDLINE(R) ALL <1946 to October 14, 2019>

Search Strategy:

--------------------------------------------------------------------------------

1 ((doctor or doctors or physician$ or medic or medics) adj4 academi$).ti,ab. (1866)

2 ((doctor or doctors or physician$ or medic or medics) adj4 (professor$ or dean$ or program$ director$ or lecturer$ or research fellow$ or researcher$)).ti,ab. (1972)

3 ((doctor or doctors or physician$ or medic or medics) adj4 (doctora$ or predoctora$ or pre-doctora$ or postdoctora$ or post-doctora$ or postdoc or post-doc or postdocs or post-docs or PhD or PhDs)).ti,ab. (230)

4 ((doctor or doctors or physician$ or medic or medics) adj4 (universit$ or higher education or research institut$ or research centre$ or research center$)).ti,ab. (1430)

5 (medical adj (profession$ or practitioner$ or specialist$) adj4 academi$).ti,ab. (63)

6 (medical adj (profession$ or practitioner$ or specialist$) adj4 (professor$ or dean$ or program$ director$ or lecturer$ or research fellow$ or researcher$)).ti,ab. (152)

7 (medical adj (profession$ or practitioner$ or specialist$) adj4 (doctora$ or predoctora$ or pre-doctora$ or postdoctora$ or post-doctora$ or postdoc or post-doc or postdocs or post-docs or PhD or PhDs)).ti,ab. (5)

8 (medical adj (profession$ or practitioner$ or specialist$) adj4 (universit$ or higher education or research institut$ or research centre$ or research center$)).ti,ab. (59)

9 ((GP or GPs or general practioner$) adj4 academi$).ti,ab. (75)

10 ((GP or GPs or general practioner$) adj4 (professor$ or dean$ or program$ director$ or lecturer$ or research fellow$ or researcher$)).ti,ab. (95)

11 ((GP or GPs or general practioner$) adj4 (doctora$ or predoctora$ or pre-doctora$ or postdoctora$ or post-doctora$ or postdoc or post-doc or postdocs or post-docs or PhD or PhDs)).ti,ab. (4)

12 ((GP or GPs or general practioner$) adj4 (universit$ or higher education or research institut$ or research centre$ or research center$)).ti,ab. (60)

13 ((dentist or dentists) adj4 academi$).ti,ab. (64)

14 ((dentist or dentists) adj4 (professor$ or dean$ or program$ director$ or lecturer$ or research fellow$ or researcher$)).ti,ab. (73)

15 ((dentist or dentists) adj4 (doctora$ or predoctora$ or pre-doctora$ or postdoctora$ or post-doctora$ or postdoc or post-doc or postdocs or post-docs or PhD or PhDs)).ti,ab. (20)

16 ((dentist or dentists) adj4 (universit$ or higher education or research institut$ or research centre$ or research center$)).ti,ab. (73)

17 ((dental or dentistry) adj (profession$ or practitioner$ or specialist$) adj4 academi$).ti,ab. (24)

18 ((dental or dentistry) adj (profession$ or practitioner$ or specialist$) adj4 (professor$ or dean$ or program$ director$ or lecturer$ or research fellow$ or researcher$)).ti,ab. (22)

19 ((dental or dentistry) adj (profession$ or practitioner$ or specialist$) adj4 (doctora$ or predoctora$ or pre-doctora$ or postdoctora$ or post-doctora$ or postdoc or post-doc or postdocs or post-docs or PhD or PhDs)).ti,ab. (1)

20 ((dental or dentistry) adj (profession$ or practitioner$ or specialist$) adj4 (universit$ or higher education or research institut$ or research centre$ or research center$)).ti,ab. (23)

21 or/1-20 (6126)

22 ((consultant$ or registrar$ or associate specialist$ or staff grade$ or house officer$ or houseman or housemen or housestaff) adj4 academi$).ti,ab. (140)

23 ((consultant$ or registrar$ or associate specialist$ or staff grade$ or house officer$ or houseman or housemen or housestaff) adj4 (professor$ or dean$ or program$ director$ or lecturer$ or research fellow$ or researcher$)).ti,ab. (240)

24 ((consultant$ or registrar$ or associate specialist$ or staff grade$ or house officer$ or houseman or housemen or housestaff) adj4 (doctora$ or predoctora$ or pre-doctora$ or postdoctora$ or post-doctora$ or postdoc or post-doc or postdocs or post-docs or PhD or PhDs)).ti,ab. (18)

25 ((consultant$ or registrar$ or associate specialist$ or staff grade$ or house officer$ or houseman or housemen or housestaff) adj4 (universit$ or higher education or research institut$ or research centre$ or research center$)).ti,ab. (166)

26 ((medical or specialt$ or specialist$ or clinical or surgical) adj4 train$ adj4 academi$).ti,ab. (445)

27 ((medical or specialt$ or specialist$ or clinical or surgical) adj4 train$ adj4 (professor$ or dean$ or program$ director$ or lecturer$ or research fellow$ or researcher$)).ti,ab. (186)

28 ((medical or specialt$ or specialist$ or clinical or surgical) adj4 train$ adj4 (doctora$ or predoctora$ or pre-doctora$ or postdoctora$ or post-doctora$ or postdoc or post-doc or postdocs or post-docs or PhD or PhDs)).ti,ab. (115)

29 ((medical or specialt$ or specialist$ or clinical or surgical) adj4 train$ adj4 (universit$ or higher education or research institut$ or research centre$ or research center$)).ti,ab. (469)

30 ((FY1 or FY2 or SHO or JHO or FY train$ or CMT or CST) adj10 academi$).ti,ab. (4)

31 ((FY1 or FY2 or SHO or JHO or FY train$ or CMT or CST) adj10 (professor$ or dean$ or program$ director$ or lecturer$ or research fellow$ or researcher$)).ti,ab. (20)

32 ((FY1 or FY2 or SHO or JHO or FY train$ or CMT or CST) adj10 (doctora$ or predoctora$ or pre-doctora$ or postdoctora$ or post-doctora$ or postdoc or post-doc or postdocs or post-docs or PhD or PhDs)).ti,ab. (0)

33 ((FY1 or FY2 or SHO or JHO or FY train$ or CMT or CST) adj10 (universit$ or higher education or research institut$ or research centre$ or research center$)).ti,ab. (31)

34 or/22-33 (1772)

35 (facult$ adj5 (medical or medicine or dental or dentistry or clinical) adj5 (academi$ or research$ or scholar$)).ti,ab. (1308)

36 (facult$ adj5 (medical or medicine or dental or dentistry or clinical) adj5 (professor$ or dean$ or program$ director$ or lecturer$ or research fellow$ or researcher$)).ti,ab. (399)

37 (facult$ adj5 (medical or medicine or dental or dentistry or clinical) adj5 (doctora$ or predoctora$ or pre-doctora$ or postdoctora$ or post-doctora$ or postdoc or post-doc or postdocs or post-docs or PhD or PhDs)).ti,ab. (79)

38 or/35-37 (1700)

39 exp Physicians/ (133162)

40 exp Dentists/ (18451)

41 Faculty, Medical/ (12740)

42 Faculty, Dental/ (2393)

43 Academic Medical Centers/ (17794)

44 39 or 40 or 41 or 42 or 43 (180173)

45 Research Personnel/ (15629)

46 Universities/ (38802)

47 research/ or biomedical research/ or dental research/ (267472)

48 45 or 46 or 47 (312512)

49 44 and 48 (7078)

50 21 or 34 or 38 or 49 (15919)

51 (academic adj (medicine or dentistry or primary care)).ti,ab. (2560)

52 (academic adj2 (an?esthesi$ or an?estheti$ or oncolog$ or emergency medicine or radiolog$ or intensive care or intensivist$ or obstetric$ or gyn?ecolog$ or ophthalmolog$ or paediatric$ or pediatric$ or patholog$ or psychiatr$ or public health or surgery or surgeon$)).ti,ab. (4925)

53 51 or 52 (7376)

54 ((clinical or clinician$ or medical or dental or dentistry) adj academi$).ti,ab. (775)

55 ((clinical or clinician$ or medical or dental or dentistry) adj (lecturer$ or lectureship$)).ti,ab. (61)

56 ((clinical or clinician$ or medical or dental or dentistry) adj professor$).ti,ab. (177)

57 ((clinical or clinician$ or medical or dental or dentistry) adj fellow$).ti,ab. (327)

58 ((clinical or clinician$ or medical or dental or dentistry) adj research fellow$).ti,ab. (37)

59 in-practice fellow$.ti,ab. (8)

60 clinical research train$.ti,ab. (102)

61 physician$ scientist$.ti,ab. (854)

62 surgeon$ scientist$.ti,ab. (163)

63 ((clinical or clinician$) adj scientist$).ti,ab. (1190)

64 ((clinical or clinician$) adj scholar$).ti,ab. (175)

65 ((clinical or clinician$) adj researcher$).ti,ab. (2576)

66 ((clinical or clinician$) adj investigator$).ti,ab. (1646)

67 ((clinical or clinician$) adj educator$).ti,ab. (1009)

68 (integrated adj3 academic adj3 (train$ or career$ or path$ or program$)).ti,ab. (33)

69 (IAT adj2 (career$ or path$ or program$)).ti,ab. (10)

70 Clinical Research Training Fellowship$.ti,ab. (10)

71 Academic Foundation Program$.ti,ab. (15)

72 (academi$ adj3 (clinical or clinician$ or medical or medicine or dental or dentistry) adj3 (career$ or path or paths or pathway$)).ti,ab. (384)

73 (research$ adj3 (clinical or clinician$ or medical or medicine or dental or dentistry) adj3 (career$ or path or paths or pathway$)).ti,ab. (329)

74 or/54-73 (9426)

75 50 or 53 or 74 (30321)

76 exp animals/ not humans/ (4628072)

77 75 not 76 (30157)

78 limit 77 to english language (27406)

79 limit 78 to yr="2004 -Current" (18838)

80 (editorial or letter).pt. (1551217)

81 79 not 80 (17633)
