# Supplementary material for: From the sticky floor to the glass ceiling and everything in between: protocol for a systematic review of barriers and facilitators to clinical academic careers and interventions to address these, with a focus on gender inequality
Source: Syst Rev. 2020 Feb 10;9:26. doi: 10.1186/s13643-020-1286-z (PMC7011470; doi:10.1186/s13643-020-1286-z)
Supplement: Supplementary file 3 — Additional file 3. Supplementary search strategy for Ovid MEDLINE. [file 13643_2020_1286_MOESM3_ESM.docx]

### Additional file 3: Supplementary search strategy

Database: Ovid MEDLINE(R) ALL <1946 to October 21, 2019>

22/10/2019

Search Strategy:

--------------------------------------------------------------------------------

1 ((doctor or doctors or physician$ or medic or medics) adj4 academi$).ti,ab. (1872)

2 ((doctor or doctors or physician$ or medic or medics) adj4 (professor$ or dean$ or program$ director$ or lecturer$ or research fellow$ or researcher$)).ti,ab. (1977)

3 ((doctor or doctors or physician$ or medic or medics) adj4 (doctora$ or predoctora$ or pre-doctora$ or postdoctora$ or post-doctora$ or postdoc or post-doc or postdocs or post-docs or PhD or PhDs)).ti,ab. (230)

4 ((doctor or doctors or physician$ or medic or medics) adj4 (universit$ or higher education or research institut$ or research centre$ or research center$)).ti,ab. (1433)

5 (medical adj (profession$ or practitioner$ or specialist$) adj4 academi$).ti,ab. (64)

6 (medical adj (profession$ or practitioner$ or specialist$) adj4 (professor$ or dean$ or program$ director$ or lecturer$ or research fellow$ or researcher$)).ti,ab. (153)

7 (medical adj (profession$ or practitioner$ or specialist$) adj4 (doctora$ or predoctora$ or pre-doctora$ or postdoctora$ or post-doctora$ or postdoc or post-doc or postdocs or post-docs or PhD or PhDs)).ti,ab. (5)

8 (medical adj (profession$ or practitioner$ or specialist$) adj4 (universit$ or higher education or research institut$ or research centre$ or research center$)).ti,ab. (59)

9 ((GP or GPs or general practioner$) adj4 academi$).ti,ab. (75)

10 ((GP or GPs or general practioner$) adj4 (professor$ or dean$ or program$ director$ or lecturer$ or research fellow$ or researcher$)).ti,ab. (95)

11 ((GP or GPs or general practioner$) adj4 (doctora$ or predoctora$ or pre-doctora$ or postdoctora$ or post-doctora$ or postdoc or post-doc or postdocs or post-docs or PhD or PhDs)).ti,ab. (4)

12 ((GP or GPs or general practioner$) adj4 (universit$ or higher education or research institut$ or research centre$ or research center$)).ti,ab. (59)

13 ((dentist or dentists) adj4 academi$).ti,ab. (63)

14 ((dentist or dentists) adj4 (professor$ or dean$ or program$ director$ or lecturer$ or research fellow$ or researcher$)).ti,ab. (73)

15 ((dentist or dentists) adj4 (doctora$ or predoctora$ or pre-doctora$ or postdoctora$ or post-doctora$ or postdoc or post-doc or postdocs or post-docs or PhD or PhDs)).ti,ab. (20)

16 ((dentist or dentists) adj4 (universit$ or higher education or research institut$ or research centre$ or research center$)).ti,ab. (72)

17 ((dental or dentistry) adj (profession$ or practitioner$ or specialist$) adj4 academi$).ti,ab. (24)

18 ((dental or dentistry) adj (profession$ or practitioner$ or specialist$) adj4 (professor$ or dean$ or program$ director$ or lecturer$ or research fellow$ or researcher$)).ti,ab. (22)

19 ((dental or dentistry) adj (profession$ or practitioner$ or specialist$) adj4 (doctora$ or predoctora$ or pre-doctora$ or postdoctora$ or post-doctora$ or postdoc or post-doc or postdocs or post-docs or PhD or PhDs)).ti,ab. (1)

20 ((dental or dentistry) adj (profession$ or practitioner$ or specialist$) adj4 (universit$ or higher education or research institut$ or research centre$ or research center$)).ti,ab. (23)

21 or/1-20 (6138)

22 ((consultant$ or registrar$ or associate specialist$ or staff grade$ or house officer$ or houseman or housemen or housestaff) adj4 academi$).ti,ab. (139)

23 ((consultant$ or registrar$ or associate specialist$ or staff grade$ or house officer$ or houseman or housemen or housestaff) adj4 (professor$ or dean$ or program$ director$ or lecturer$ or research fellow$ or researcher$)).ti,ab. (243)

24 ((consultant$ or registrar$ or associate specialist$ or staff grade$ or house officer$ or houseman or housemen or housestaff) adj4 (doctora$ or predoctora$ or pre-doctora$ or postdoctora$ or post-doctora$ or postdoc or post-doc or postdocs or post-docs or PhD or PhDs)).ti,ab. (18)

25 ((consultant$ or registrar$ or associate specialist$ or staff grade$ or house officer$ or houseman or housemen or housestaff) adj4 (universit$ or higher education or research institut$ or research centre$ or research center$)).ti,ab. (166)

26 ((medical or specialt$ or specialist$ or clinical or surgical) adj4 train$ adj4 academi$).ti,ab. (444)

27 ((medical or specialt$ or specialist$ or clinical or surgical) adj4 train$ adj4 (professor$ or dean$ or program$ director$ or lecturer$ or research fellow$ or researcher$)).ti,ab. (186)

28 ((medical or specialt$ or specialist$ or clinical or surgical) adj4 train$ adj4 (doctora$ or predoctora$ or pre-doctora$ or postdoctora$ or post-doctora$ or postdoc or post-doc or postdocs or post-docs or PhD or PhDs)).ti,ab. (115)

29 ((medical or specialt$ or specialist$ or clinical or surgical) adj4 train$ adj4 (universit$ or higher education or research institut$ or research centre$ or research center$)).ti,ab. (468)

30 ((FY1 or FY2 or SHO or JHO or FY train$ or CMT or CST) adj10 academi$).ti,ab. (4)

31 ((FY1 or FY2 or SHO or JHO or FY train$ or CMT or CST) adj10 (professor$ or dean$ or program$ director$ or lecturer$ or research fellow$ or researcher$)).ti,ab. (20)

32 ((FY1 or FY2 or SHO or JHO or FY train$ or CMT or CST) adj10 (doctora$ or predoctora$ or pre-doctora$ or postdoctora$ or post-doctora$ or postdoc or post-doc or postdocs or post-docs or PhD or PhDs)).ti,ab. (0)

33 ((FY1 or FY2 or SHO or JHO or FY train$ or CMT or CST) adj10 (universit$ or higher education or research institut$ or research centre$ or research center$)).ti,ab. (31)

34 or/22-33 (1771)

35 (facult$ adj5 (medical or medicine or dental or dentistry or clinical) adj5 (academi$ or research$ or scholar$)).ti,ab. (1311)

36 (facult$ adj5 (medical or medicine or dental or dentistry or clinical) adj5 (professor$ or dean$ or program$ director$ or lecturer$ or research fellow$ or researcher$)).ti,ab. (399)

37 (facult$ adj5 (medical or medicine or dental or dentistry or clinical) adj5 (doctora$ or predoctora$ or pre-doctora$ or postdoctora$ or post-doctora$ or postdoc or post-doc or postdocs or post-docs or PhD or PhDs)).ti,ab. (79)

38 or/35-37 (1703)

39 exp Physicians/ (133470)

40 exp Dentists/ (18469)

41 Faculty, Medical/ (12749)

42 Faculty, Dental/ (2396)

43 Academic Medical Centers/ (17825)

44 39 or 40 or 41 or 42 or 43 (180534)

45 Research Personnel/ (15653)

46 Universities/ (38888)

47 research/ or biomedical research/ or dental research/ (267736)

48 45 or 46 or 47 (312870)

49 44 and 48 (7088)

50 21 or 34 or 38 or 49 (15943)

51 (academic adj (medicine or dentistry or primary care)).ti,ab. (2568)

52 (academic adj2 (an?esthesi$ or an?estheti$ or oncolog$ or emergency medicine or radiolog$ or intensive care or intensivist$ or obstetric$ or gyn?ecolog$ or ophthalmolog$ or paediatric$ or pediatric$ or patholog$ or psychiatr$ or public health or surgery or surgeon$)).ti,ab. (4935)

53 51 or 52 (7393)

54 ((clinical or clinician$ or medical or dental or dentistry) adj academi$).ti,ab. (778)

55 ((clinical or clinician$ or medical or dental or dentistry) adj (lecturer$ or lectureship$)).ti,ab. (61)

56 ((clinical or clinician$ or medical or dental or dentistry) adj professor$).ti,ab. (177)

57 ((clinical or clinician$ or medical or dental or dentistry) adj fellow$).ti,ab. (325)

58 ((clinical or clinician$ or medical or dental or dentistry) adj research fellow$).ti,ab. (37)

59 in-practice fellow$.ti,ab. (8)

60 clinical research train$.ti,ab. (103)

61 physician$ scientist$.ti,ab. (854)

62 surgeon$ scientist$.ti,ab. (166)

63 ((clinical or clinician$) adj scientist$).ti,ab. (1193)

64 ((clinical or clinician$) adj scholar$).ti,ab. (175)

65 ((clinical or clinician$) adj researcher$).ti,ab. (2590)

66 ((clinical or clinician$) adj investigator$).ti,ab. (1648)

67 ((clinical or clinician$) adj educator$).ti,ab. (1010)

68 or/54-67 (8881)

69 50 or 53 or 68 (30068)

70 (integrated adj3 academic adj3 (train$ or career$ or path or paths or pathway$ or program$)).ti,ab. (33)

71 (IAT adj2 (career$ or path$ or program$)).ti,ab. (10)

72 Clinical Research Training Fellowship$.ti,ab. (10)

73 Academic Foundation Program$.ti,ab. (15)

74 (academi$ adj3 (clinical or clinician$ or medical or medicine or dental or dentistry) adj3 (career$ or path or paths or pathway$)).ti,ab. (384)

75 (research$ adj3 (clinical or clinician$ or medical or medicine or dental or dentistry) adj3 (career$ or path or paths or pathway$)).ti,ab. (330)

76 or/70-75 (750)

77 Career Choice/ (22455)

78 career mobility/ (11221)

79 Staff Development/ (9135)

80 (career$ or pathway$ or pipeline$).ti,ab. (1104701)

81 ((occupation$ or profession$ or job$ or staff or employee$) adj3 (choice$ or choos$ or select$ or decide$ or decision$)).ti,ab. (6851)

82 ((occupation$ or profession$ or job$ or staff or employee$) adj3 (mobility$ or ladder$ or route$ or trajector$ or structure$)).ti,ab. (2732)

83 ((occupation$ or profession$ or job$ or staff or employee$) adj3 (progress$ or promot$ or develop$ or advanc$)).ti,ab. (25660)

84 77 or 78 or 79 or 80 or 81 or 82 or 83 (1163283)

85 69 and 84 (4284)

86 Personnel Selection/ (12616)

87 (recruit$ or hire$ or hiring).ti,ab. (354925)

88 86 or 87 (363105)

89 69 and 88 (1456)

90 Personnel Turnover/ (5019)

91 (retain$ or retention).ti,ab. (350492)

92 (resign$ or terminat$ or disenroll$ or withdraw$ or attrition).ti,ab. (247045)

93 90 or 91 or 92 (595056)

94 69 and 93 (885)

95 85 or 89 or 94 (5695)

96 76 or 95 (6022)

97 exp animals/ not humans/ (4635158)

98 96 not 97 (6011)

99 limit 98 to english language (5713)

100 limit 99 to yr="2004 -Current" (4447)

101 (editorial or letter).pt. (1553117)

102 100 not 101 (4217)

### 
