# Supplementary material for: From the sticky floor to the glass ceiling and everything in between: protocol for a systematic review of barriers and facilitators to clinical academic careers and interventions to address these, with a focus on gender inequality
Source: Syst Rev. 2020 Feb 10;9:26. doi: 10.1186/s13643-020-1286-z (PMC7011470; doi:10.1186/s13643-020-1286-z)
Supplement: Supplementary file 4 — Additional file 4. Data extraction form. [file 13643_2020_1286_MOESM4_ESM.docx]

### Additional file 4: Data extraction form

#### General information

Person performing data extraction:

Date of data extraction:

Person checking data extraction:

Date of checking:

Record number:

Study title:

Study Author, Year:

Country (or countries) in which research was performed:

Source of funding:

Type of publication: Full text ☐ Conference Abstract only ☐

#### Study Information

Stated aim of study:

Quantitative ☐ Qualitative ☐

Study design:

Risk of bias tool completed: Cochrane risk of bias tool ☐ Newcastle Ottawa ☐ QARI ☐

Inclusion criteria:

Exclusion criteria:

Definition of clinical academic used by study:

Intervention (if present, as described by study, to include as much detail as possible, in order to allow for evaluation of the elements contributing to the findings):

Comparator/control group (if present):

Details of randomisation/selection of cohorts:

#### Participants

Number of participants:

Number in each group:

Number withdrawn:

Number included in analysis:

Age – provide details for each group:

Sex – provide details for each group:

Ethnicity (if given):

Professional group (doctor/dentist/other; clinical speciality/specialities; proportion of academics):

Stage of career (if given, for either clinical or academic or both):

Other important population factors (eg those with parental responsibilities only):

Are recruitment/refusal to consent numbers given? If so, please record details including, if given, number, distribution, reasons for declining:

#### Outcomes

Section of review to be included in:

Barriers and facilitators ☐

Interventions ☐

Primary outcome(s), including definition of each, as given by the study (to be consistent with outcomes defined in the methods section of this protocol):

Secondary outcome(s), including definition of each, as given by the study (to be consistent with outcomes defined in the methods section of this protocol):
